# Offloading items from memory: individual differences in cognitive offloading in a short-term memory task

**DOI:** 10.1186/s41235-019-0201-4

**Published:** 2020-01-03

**Authors:** Alexandra B. Morrison, Lauren L. Richmond

**Affiliations:** 1 Department of Psychology, California State University, Sacramento, 6000 J Street, Sacramento, CA USA; 2 Department of Psychology, Stony Brook University, Psychology B Building, Stony Brook, NY 11794 USA

**Keywords:** Cognitive offloading, Working memory capacity, Strategy use

## Abstract

Cognitive offloading refers to the act of reducing the mental processing requirements of a task through physical actions like writing down information or storing information on a cell phone or computer. Offloading can lead to improved performance on ongoing tasks with high cognitive demand, such as tasks where multiple pieces of information must be simultaneously maintained. However, less is known about why some individuals choose to engage in offloading and under what conditions they might choose to do so. In the present study, offloading behavior is investigated in a short-term memory task requiring memory for letters. The present study is a replication and extension of a previous study conducted by Risko and Dunn, and tests the new prediction that individuals with lower working memory capacity will be more likely to offload. Here, we find that offloading information confers a performance advantage over relying on internal memory stores, particularly at higher memory loads. However, we fail to observe that those with poorer memory abilities have a greater propensity for offloading or benefit more from it. Instead, our findings suggest that cognitive offloading may be a valid compensatory strategy to improve performance of memory-based tasks for individuals with a wide range of memory ability.

## Significance

During daily life, it is common to encounter situations where multiple pieces of information must be kept in mind at once. One strategy for succeeding in such situations is to engage in cognitive offloading by writing information down or storing it digitally. The present study examines the likelihood of cognitive offloading in a short-term memory task with a focus on the characteristics of individuals who choose to offload. This work is relevant for both remediation strategies that can be used for tasks that would normally demand a high mental load as well as for individuals who struggle with high-load situations.

## Introduction

If you have ever made a grocery list, programmed an appointment into your online calendar, or used your calculator to figure out the appropriate tip on a restaurant bill, you have used cognitive offloading as a strategy in your daily life. Cognitive offloading is defined as “the use of physical action to alter the information processing requirements of a task so as to reduce cognitive demand” (Risko & Gilbert, 2016, p. 677). Offloading can help overcome the well-established capacity limits of cognitive processes such as working memory or visual perception and has been shown to reliably improve performance in these domains compared to conditions in which offloading is prohibited (see Risko & Gilbert, 2016, for a review). This type of behavior is something that many of us engage in throughout the course of our daily lives.

Despite the prevalence of cognitive offloading in modern life, this type of behavior has been studied very little in comparison to the wealth of literature dedicated to investigating facets of internal information storage (i.e., storing mentally). Here, we seek to better characterize the individuals who tend to offload versus those that more often rely on their internal memory stores. We specifically examine whether one’s propensity for offloading information is tied to their working memory capacity (WMC), defined broadly as the number of items one can simultaneously hold in a highly accessible state (Cowan, 2017). We test the prediction that those who can hold fewer items in working memory will be more likely to offload.

### Previous work on cognitive offloading

In recent years, the nascent field of cognitive offloading has examined two main questions: 1) what factors influence whether one chooses to offload; and 2) what are the downstream cognitive consequences of choosing to engage in offloading (Risko & Gilbert, 2016)?

To address the first question, researchers have assessed both traits and tendencies within individuals that might predict the likelihood of offloading. Recent work has also experimentally manipulated conditions under which participants may be more inclined to engage in offloading. Perhaps unsurprisingly, lower objective memory ability and lower subjective memory ratings corresponded to higher levels of offloading behavior (Gilbert, 2015b). More interestingly, these patterns held true even when subjective confidence was not correlated with objective memory performance (Gilbert, 2015b). Recent work by Risko and Dunn (2015) replicated these findings in a short-term memory task involving letter memory. They demonstrated that short-term memory capacity, measured by performance in a “no-choice condition” of their task where participants were not permitted to offload, was inversely related to the frequency of offloading in the choice condition during which participants were able to choose whether or not to offload information. This finding regarding short-term memory capacity and offloading will be further investigated herein.

Moving on to experimental manipulations that influence offloading behaviors, researchers have identified two main factors that make offloading more likely. Gilbert (2015a) administered a task that involved dragging numbered circles to the bottom of the screen in order from 1 to 10, except for the instruction to move either one or three circles to different locations on the screen. The tendency to choose to engage in offloading was influenced by increased memory load (three-circle condition compared to one-circle condition) or the addition of a secondary processing demand to the ongoing task (in this case completing a distractor task during the trial). In short, increasing the memory demands of the task and/or reducing available attentional resources that can be devoted to the task at hand made participants more likely to engage in offloading behavior.

With respect to the question of the downstream effects of offloading, as one might suspect, offloaded intentions are more likely to be fulfilled later compared to intentions that are not offloaded (Gilbert, 2015a, 2015b), suggesting an overall benefit for offloaded versus internally stored representations. However, in some cases individuals choose to offload even when it does not improve performance (i.e., when performance is already at ceiling; Gilbert, 2015a, 2015b; Risko & Dunn, 2015). Why might individuals choose to exert the effort to offload without an obvious performance benefit? Recent work suggests that individuals who engage in offloading even when there is no objective performance benefit may be driven to do so by the incorrect belief that offloading will lead to better performance (Risko & Dunn, 2015).

There are also some downsides to choosing to offload. In an experiment where participants studied trivia statements and believed that the studied information would or would not be stored for later access (here, believing that the statements would be stored and accessible later is thought of as akin to offloading), participants who thought they would have access to the stored information later showed poorer memory for the studied items (Sparrow, Liu, & Wegner, 2011). Similar effects have been found with visual stimuli. In a study where participants were asked to visit a museum and take photos of some artifacts and simply visit and observe other artifacts, those objects that were photographed were remembered more poorly later. This has been termed the photo-taking-impairment effect (Henkel, 2014). More recent work suggests that this effect may not be solely due to the belief that the camera will do the ‘remembering’ for them; recently, the photo-taking-impairment effect was observed even when participants did not believe they would have access to photos later (Soares & Storm, 2017). The authors of this recent work suggest that engaging in photo-taking might disrupt the typical processing or encoding of object features (Soares & Storm, 2017), but more work is necessary to disentangle the consequences of engaging in offloading for processes related to initial encoding and for subsequent recall of information.

These prior findings suggest that offloading can lead to impairments in performance when offloaded information is not available at the time the information is needed, but that offloaded information, when available for use later, typically benefits performance. In addition, lower subjective ratings of memory ability and greater cognitive demand during the task have been found to relate to higher levels of offloading behavior. However, less is known about the specific cognitive processes that may be involved in or disrupted by offloading.

### Individual differences in WMC and relation to other cognitive domains

Working memory was recently described as reflecting “an ability to maintain information in the maelstrom of divergent thought” (Engle, 2018, p. 192), and we predict that the capacity of this cognitive system may relate to whether one offloads information for future use as a memory aid. However, extant models of working memory and methods of testing WMC do not consider or test whether, when given the opportunity, people offload information from working memory to external sources. This limits the extent to which these models apply to real-world situations in which individuals use external memory aids.

The present work bridges the study of cognitive offloading with the literature examining individual differences in WMC. WMC has long been recognized as related to performance in other cognitive domains. For example, individuals with higher WMC show better performance on reasoning tasks (Kane et al., 2004), reading comprehension (Daneman & Carpenter, 1980) and controlled search of long-term memory (Unsworth, Brewer, & Spillers, 2013). In addition, people with higher WMC estimates exhibit smaller Stroop interference effects (Kane & Engle, 2003), reduced interference from background noise (Conway, Cowan, & Bunting, 2001), and the tendency to deploy proactive cognitive control (Redick, 2014; Richmond, Redick, & Braver, 2015; Wiemers & Redick, 2018). However, individuals with high WMC are not universally advantaged, and deficits have also been associated with higher WMC. Individuals with higher WMC have more difficulty recovering items in a directed forgetting task (Delaney & Sahakyan, 2007), exhibit poorer recognition memory on a surprise recall test for neutral words in a Stroop task (Shipstead & Broadway, 2013), and show longer response times on trials for which participants must override the prepotent response in a cognitive control task (Richmond et al., 2015).

While robust in many areas, the literature on individual differences in WMC says less about whether high and low WMC individuals differ in their tendency to store information internally (using working memory) versus externally (by offloading information to the external world). As WMC is operationalized as performance on measures of working memory (e.g., operation span; Unsworth, Heitz, Schrock, & Engle, 2005), high-span individuals excel by demonstrating that they *can* successfully retain multiple memory items over the short term. However, the tasks used to measure WMC do not typically afford the opportunity to offload, and just because individuals *can* store multiple items in working memory does not mean that they *will* when given an alternative (i.e., writing an item down). A primary aim of the present work is to investigate whether WMC is related to how often one offloads during a short-term memory task (when given the choice).

If there is indeed a relationship between offloading and capacity, one could predict that those who can remember more would offload less. Or, it could be that those with higher capacity also excel at strategic allocation of resources and therefore might be able to identify when offloading may be useful. Risko and Dunn (2015) showed evidence for the former; those who performed better on a verbal short-term memory task were less likely to offload when given the opportunity. The present study seeks to replicate and extend this work by using an expanded battery of tasks to measure WMC on a construct level (i.e., with the aggregate of performance on multiple tasks). This approach will allow for measurement of an underlying WMC construct, and will use complex span working memory measures that are common to the individual differences in WMC literature (Conway et al., 2005). This approach is in line with recent studies focused on individual differences in WMC and its relation to cognitive functioning in other domains (e.g., Redick, Calvo, Gay, & Engle, 2011; Richmond et al., 2015; Shipstead & Broadway, 2013).

### Current research questions and hypotheses

Here, we aim to elucidate the relationship between memory ability and offloading behavior in a simple short-term memory task. In this study, we plan to assess four research questions. The first two questions are replications of work presented by Risko and Dunn (2015): 1) does having the choice to offload items from memory by writing them down lead to better performance than using memory alone; does this differ by load; and 2) within a given set size, does choosing to write in the choice condition benefit performance? For both questions 1 and 2, we expect to replicate the effects reported in Risko and Dunn (2015; experiments 1a and 1b), showing that offloading benefits performance and is more beneficial at larger set sizes.

Next, we expand upon previous work by asking: 3) does WMC predict the likelihood that one chooses to offload beyond what is already explained by performance in the no-choice block? We expect that WMC will account for additional variance in offloading frequency, and that it will do so in a negative fashion (i.e., individuals with higher WMC engage in lower levels of offloading). Finally, we examine the predicted performance advantage on trials where the participant can choose to offload (choice condition) versus trials where they must rely on internal memory (no-choice condition). We ask, 4) do WMC and frequency of offloading explain the difference between performance in the choice and no-choice conditions? This fourth question will focus on set sizes thought to be at or above the typical upper limit of WMC (set sizes 6, 8, 10), where offloading should be of benefit. We expect that WMC and the frequency of offloading will each explain unique variance in difference scores. We predict that WMC and choice minus no-choice accuracy will be inversely related to one another (i.e., higher WMC estimates will be related to smaller difference scores), whereas we expect that frequency of offloading will be positively related to choice minus no-choice performance (i.e., higher levels of offloading will predict a larger difference in choice – no-choice performance).

## Method

In the stage 1 submission of this registered report, we proposed the following protocol, which we executed as planned.

### Stimuli and task

**Fig. 1:**
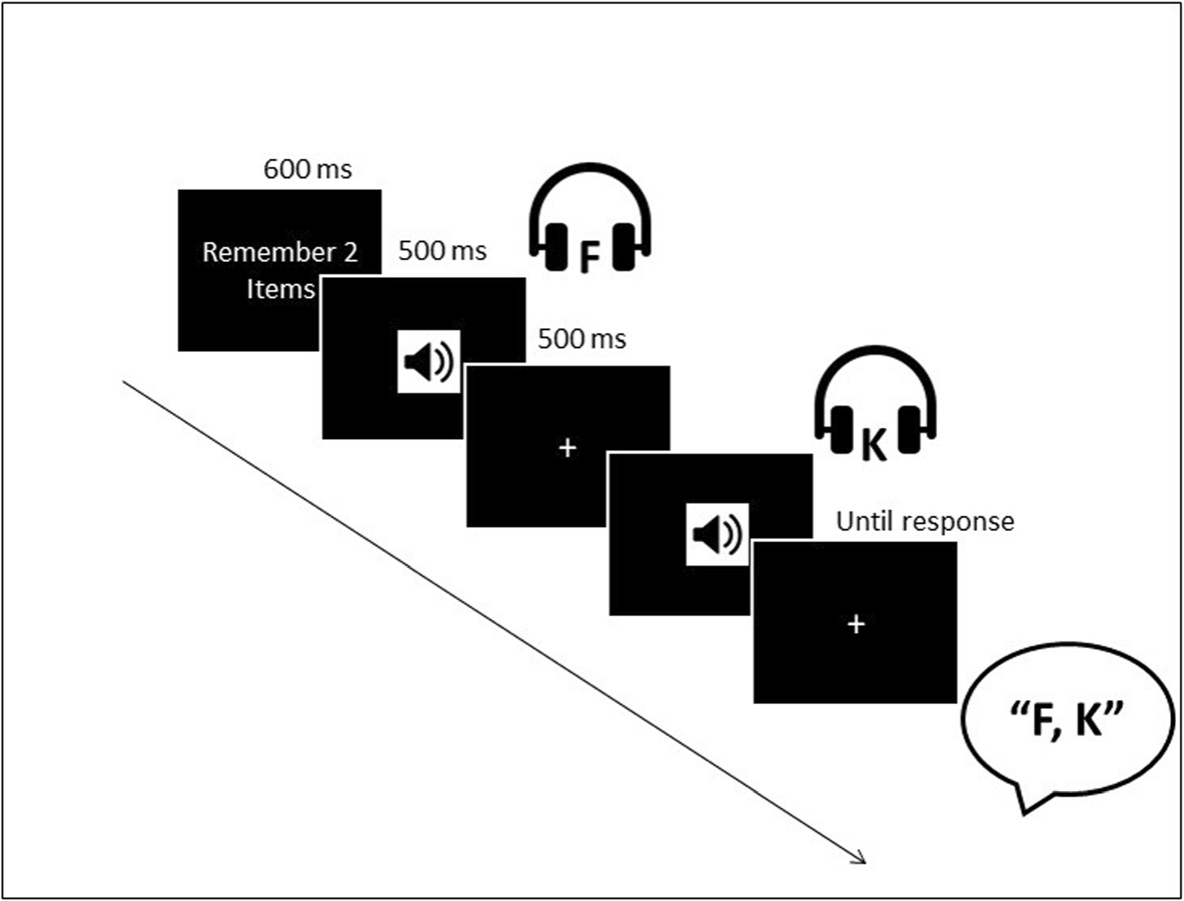
Schematic of the trial sequence for a sample trial of the short-term memory task

Participants were asked to engage in a short-term memory task similar to that described in experiment 1a by Risko and Dunn (2015). The current study used the same stimulus set (BCFHJKLMPQRTWX), set sizes (2, 4, 6, 8, 10 letters), and auditory stimulus presentation as the experiment by Risko and Dunn. The task was administered using Eprime 3.0 (Psychology Software Tools, Pittsburgh, PA, USA) Psychology Software Tools, Inc, 2016. At the start of each trial, a number appeared on screen for 600 ms alerting participants to the set size for the upcoming trial (e.g., “Remember 6 items”). Stimuli were presented auditorily at a rate of roughly one item per second, mirroring the pace of auditory stimulus presentation used by Risko and Dunn (2015; see Fig. 1 for a task schematic). The experimenter remained in the testing room and, on each trial, they recorded the participants’ responses. The sessions were also audio recorded.

During the choice condition, participants were given a paper and pen should they decide to write anything down. The experimenter recorded whether the participant wrote anything during the choice block on a trial-by-trial basis.

### Procedure

At the start of the testing session, participants were told “In this experiment you will be presented with a list of letters ranging in length from 2 to 10 letters. Your task is to remember the letters presented and to report them back when the screen says REPORT. You need to report them back IN THE ORDER THEY WERE PRESENTED. At the start of each trial a number will indicate how many letters there will be. Remember as many letters IN ORDER as possible even if you cannot get them all. This task will be difficult at times just try your best.” These instructions were also used in Risko and Dunn (2015; E. Risko, personal communication, 24 August 2017).

Following the instructions, participants completed a short practice phase with two trials each containing 2-item lists without the option to offload in order to ensure understanding of how stimuli would be presented and how they were expected to respond. After the practice phase, participants performed two blocks of trials (a no-choice block and a choice block) with 30 trials per block. In the no-choice condition, participants completed the task as a standard auditory short-term memory task. They were instructed to attend to the presented stimuli. Then, immediately after the last stimulus was presented, they were prompted to recall the items in the order they were presented. Participants reported the items by saying them out loud to the experimenter. Trials were marked as correct if all letters were reported in correct serial order.

In the choice condition, stimulus presentation and recall occurred identically to the no-choice condition, with the exception that participants had the choice to offload. Participants were told that they could choose to use the paper provided to write down anything they wished, but that they were not required to use the pen and paper if they wished not to. Participants were not provided any information about what they should write down or when they might begin writing should they choose to do so. Conditions (choice and no-choice) were blocked, and order was counterbalanced across subjects.

After the completion of the short-term memory task, participants completed two complex working memory span tasks: automated operation span and symmetry span (Unsworth et al., 2005) in that order. In brief, both tasks involve a processing component and a storage component. Operation span involves solving simple math problems interleaved with the presentation of to-be-remembered letters. Set sizes range from 3 to 7. Symmetry span involves making decisions about the left–right symmetry of a design created by filling in some squares of an 8 × 8 grid, interleaved with remembering highlighted locations on a 4 × 4 grid. Set sizes included in this task range from 2 to 5. On both tasks, participants are not told the set size prior to the trial, but they do receive feedback on both the decision portion and memory performance at the end of each trial.

### Power analysis and stopping rule

We collected full datasets (meeting all inclusion criteria) from 114 participants. This sample size was determined in an effort to power our study to investigate individual differences in short-term and working memory and their relation to offloading behavior. Power was calculated for the correlation between performance in the no-choice block of the short-term memory task and the likelihood of engaging in offloading. This correlation was chosen in order to replicate the analysis conducted by Risko and Dunn (2015). Sample size was calculated based on the smallest effect size of interest (Lakens & Evers, 2014). In this case, we were interested in powering our study to detect a medium-sized correlation. Therefore, assuming an alpha of .05 and a desired power of 90%, a sample size of 113 is required to detect whether a correlation coefficient of .300 differs from zero. In order to maintain a full counterbalance of the order of the two conditions (choice, no-choice), we added one additional participant to our sample for a final sample size of 114.^1^ We feel that any effect smaller than this would be of little theoretical import to the working memory literature and practical import in daily life.

### Statistical analyses

In the stage 1 submission of this registered report, we proposed the following analysis plan, which we executed as planned following data collection. In order to assess the effects of offloading and load (question 1), we conducted a 2 (instruction: no-choice vs. choice) × 5 (load: 2, 4, 6, 8, 10) analysis of variance (ANOVA) on accuracy and examined main effects and interactions. To mirror the analysis strategy employed by Risko and Dunn (2015), we inspected patterns visually, forgoing conducting formal contrasts. We expected to observe main effects of condition and load as well as a condition × load interaction, such that the effect of load was less pronounced in the choice condition compared to the no-choice condition, replicating the findings reported in Risko and Dunn (2015).

To examine whether choosing to write in the choice condition is beneficial to performance (question 2), we conducted a series of *t* tests at each load comparing accuracy for trials on which individuals chose to write within the choice block and overall performance at each set size in the no-choice block. Again, we expected to replicate the findings of Risko and Dunn (2015); they observed significant effects of writing at set sizes 4, 6, 8 and 10 but there was no significant difference at set size 2.

In order to assess whether memory ability contributes to the likelihood that one chooses to offload (question 3), we first ran the analysis employed by Risko and Dunn (2015). We investigated the correlation between accuracy in the no-choice condition (as a proxy for baseline memory ability) with the likelihood of engaging in offloading in the choice condition. We expected that higher accuracy in the no-choice condition would be related to less offloading behavior in the choice condition, as reported by Risko and Dunn (2015).

Next, in order to more directly address the role of WMC in offloading behavior, we ran a second analysis relating WMC to the likelihood of choosing to offload. We examined whether WMC explains variance above and beyond what is explained by performance in the no-choice block. Here, we expected that adding WMC to the model would improve variance explained in offloading behavior. We planned to conduct a stepwise regression where we predicted frequency of offloading as the dependent variable with no-choice block performance in step 1 and WMC in step 2. Our metric of WMC was created by calculating *Z* scores of each working memory task using the norms reported in Redick et al. (2012) and then averaging them to create a composite measure of WMC. This standardizing was necessary as the two tasks are on different scales with different maximum scores.

Next, we addressed the role of WMC and choice behavior in predicting the benefit to performance that resulted from offloading (question 4) by conducting a regression to predict the difference score for accuracy on the larger set sizes (6, 8 and 10) in the choice minus the no-choice condition with the idea that these set sizes in particular are where one’s WMC might play an important role in performance. We entered WMC and frequency of offloading in the choice block into the model simultaneously as predictors. To reiterate briefly, we expected that WMC and frequency of offloading would predict unique variance in the difference between choice minus no-choice performance on large set sizes. We expected that higher levels of WMC would be related to smaller differences, and that frequency of offloading would predict larger differences in choice – no-choice performance.

Prior to conducting these regressions, we checked for evidence of suppression by examining whether the part correlation of the independent variance with the dependent variable was greater (by absolute value) than the zero-order *r* between them. Had we observed evidence of suppression among the variables within the model we describe above, we would have run two separate regressions; in one, we would have entered WMC as the predictor of choice minus no-choice performance, and in a second, separate model offloading would be entered as the predictor.

### Participants

Participants consisted of a sample of university students. The following exclusion criterion were determined prior to data collection. All participants were required to be fluent in English with normal or corrected-to-normal vision and hearing. Any participant obtaining less than 50% accuracy on the processing portion of either Operation Span or Symmetry Span would be excluded. Similarly, any subject performing below 50% accuracy on set size 2 in the short-term memory task would be excluded. These data would be excluded as performance below these thresholds indicates extremely low task engagement. Participants were also given a brief questionnaire at the end of the study checking for understanding of the choice condition in the short-term memory task; any participant indicating that they did not understand the choice manipulation (i.e., thinking that they were not allowed to write in the choice condition or thinking that they were required to write in the choice condition) would be excluded. All participants consented to participate, and the study was approved by the Institutional Review Boards at California State University, Sacramento, and The State University of New York at Stony Brook.

### Data sharing

Raw data and other materials such as analysis scripts are publicly available via the Open Science Framework website: https://osf.io/aq5ft/. Auditory short-term memory task is available from the authors upon request, and WMC tasks are available by request at http://englelab.gatech.edu/taskdownloads.

## Results

Data were collected from 130 subjects total across the two sites, and data from 16 participants were excluded from the final analysis for the following reasons: did not meet stated language fluency and/or vision criteria for inclusion (*n* = 8), computer/experimenter error (*n* = 2), failure to follow and/or understand study instructions (*n* = 4), and performance below stated inclusion criteria (*n* = 2). This resulted in a final dataset of 114 participants, all of whom were undergraduate students and received course credit for their participation.

Responses on the auditory short-term memory task were recorded during the experimental session by the experimenter, as was offloading behavior. Testing sessions were audio recorded so that participants’ responses could be coded offline and inter-rater reliability between online and offline responses calculated. A research assistant who was not involved in data collection listened to and recorded responses for 24 participants whose data was included in the final sample (12 from each site), comprising just over 21% of our final dataset. Inter-rater reliability, as assessed by Cohen’s κ, was examined to assess the agreement between online and offline response coding for these 24 participants. There was very good agreement between online and offline response records, κ = .914, *p* < .001. Due to the high agreement between online and offline responses, all analyses reported below relied on responses recorded by the experimenter running each testing session (e.g., online response recording).

Following the method employed by Risko and Dunn (2015), scoring was done in an all-or-none fashion, such that participants had to respond with all of the correct letters, in the correct order, in order to receive credit for having responded correctly on a given trial.^2^

### Effects of offloading choice and load on performance

**Fig. 2:**
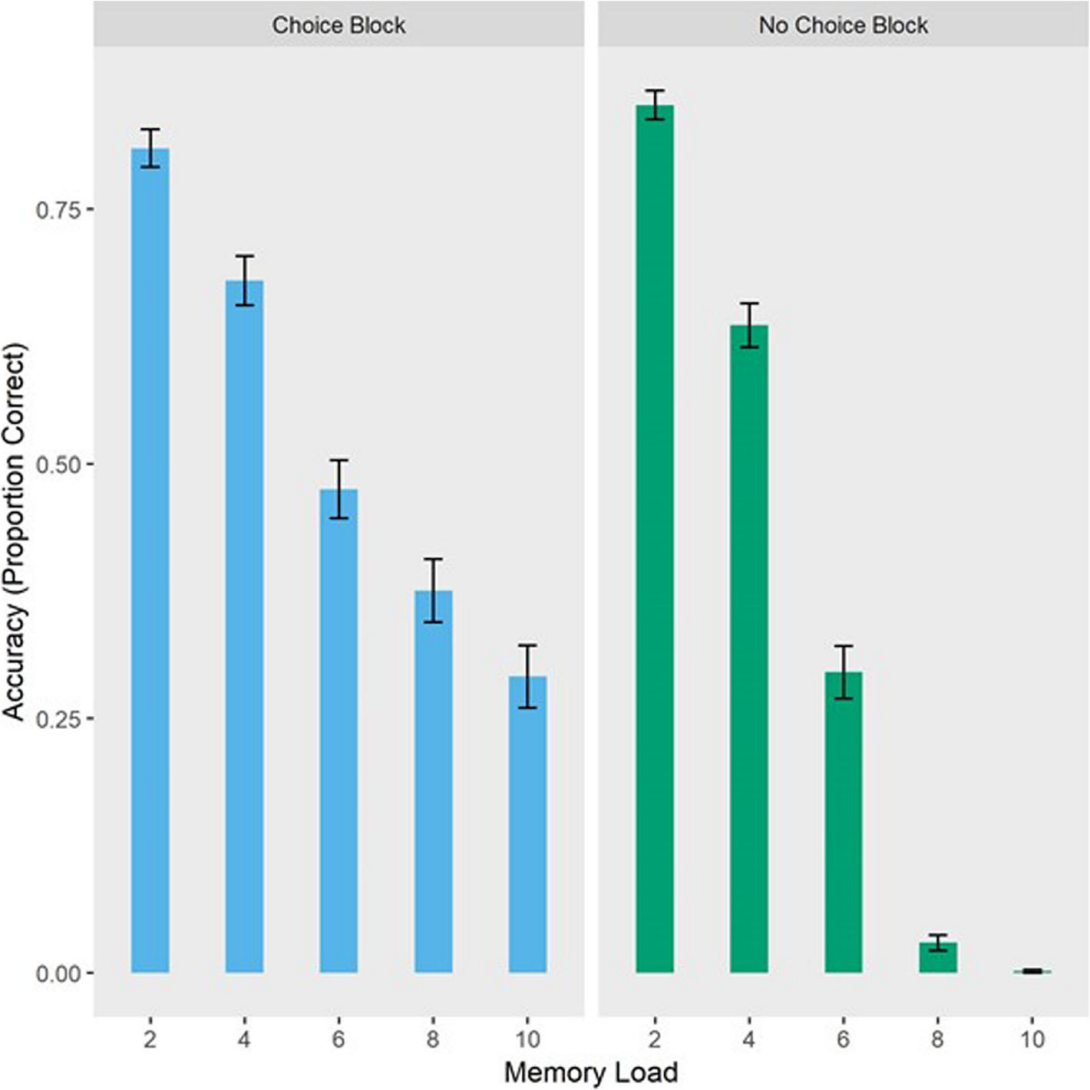
Average performance by set size in the choice block (where the participants could offload items from memory) and the no-choice block (where they had to rely on internal memory). Error bars denote standard error

**Fig. 3:**
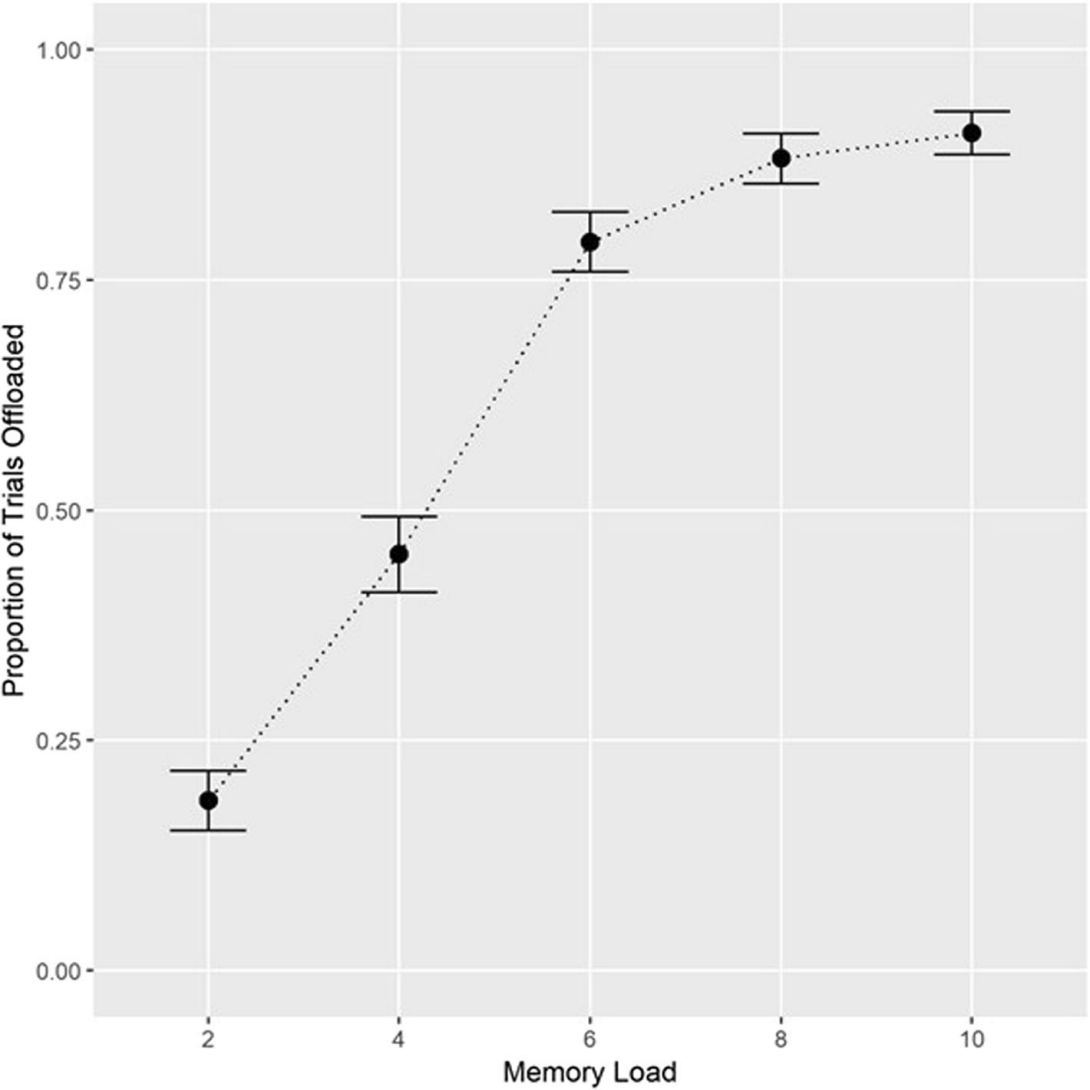
Average proportion of trials offloaded by set size in the choice block. Error bars denote standard error

To address our first research question, we conducted a 2 (condition: choice, no-choice) × 5 (load: 2, 4, 6, 8, 10 items) within-subjects ANOVA on performance. Here, we expected to replicate Risko and Dunn (2015) with a significant main effect of condition and a significant condition × load interaction. (We also expected a significant main effect of memory load, though this main effect was not directly of interest to the current study.) Using the ezANOVA package (Lawrence, 2016) in R (R Core Team, 2008), we found a significant main effect of condition (*F* (1, 113) = 96.539, *p* < .001, η^2^_G_ = 0.105) with better performance in the choice than the no-choice block and a significant main effect of memory load where performance decreased as load increased (*F* (4, 452) = 676.178, *p* < .001, η^2^_G_ = 0.549). We also found a significant condition × load interaction (*F* (4, 452) = 42.478, *p* < .001, η^2^_G_ = 0.085) where the load effect was less pronounced in the choice condition (Fig. 2). These effects replicated those reported in Risko and Dunn (2015), and supported our hypotheses. Another pattern that was consistent with the earlier findings of Risko and Dunn (2015) was that the proportion of trials offloaded increased as memory load increased (Fig. 3).

### Benefit of choice on performance at each set size

Next, following the analysis scheme from Risko and Dunn (2015), we conducted a *t* test at each memory load comparing average accuracy for offloaded trials and average performance at that set size for all trials in the no-choice block. These analyses were necessarily limited to individuals who offloaded at least one trial in the given load. Again, we expected to replicate Risko and Dunn (2015) with significant effects of offloading at set sizes greater than 2 (and no difference with only 2 items).

Contrary to our hypothesized null effect at 2 items, we see a significant advantage for no-choice performance compared to performance on offloaded trials at set size 2 (*t* (38) *=* −2.647, *p* = .012, *d* = −0.535). It should be noted, however, that this effect is also against the predicted advantage for performance on choice trials and, due to the fact that this analysis is limited only to participants who chose to offload at least once at a set size of 2, includes only a small subset of our data. Turning to a memory load of 4 items, we do not observe a difference between performance on trials for which participants chose to offload versus relying on internal memory in the no-choice block (*t* (68) = 0.910, *p* = .366, *d* = 0.115). Thus, findings at set sizes 2 and 4 failed to replicate Risko and Dunn (2015) and did not provide support for our hypotheses. However, at set sizes of 6 (*t* (100) = 6.278, *p* < .001, *d* = 0.729), 8 (*t* (104) = 12.113, *p* < .001, *d* = 1.478), and 10 (*t* (107) = 9.672, *p* < .001, *d* = 1.312) the predicted pattern did emerge where there was a performance advantage on offloaded trials compared to when offloading was not an available strategy. Together, these results provide some preliminary evidence for the performance advantage afforded by offloading at higher memory loads.

### Short-term memory performance and offloading choice behavior

**Fig. 4:**
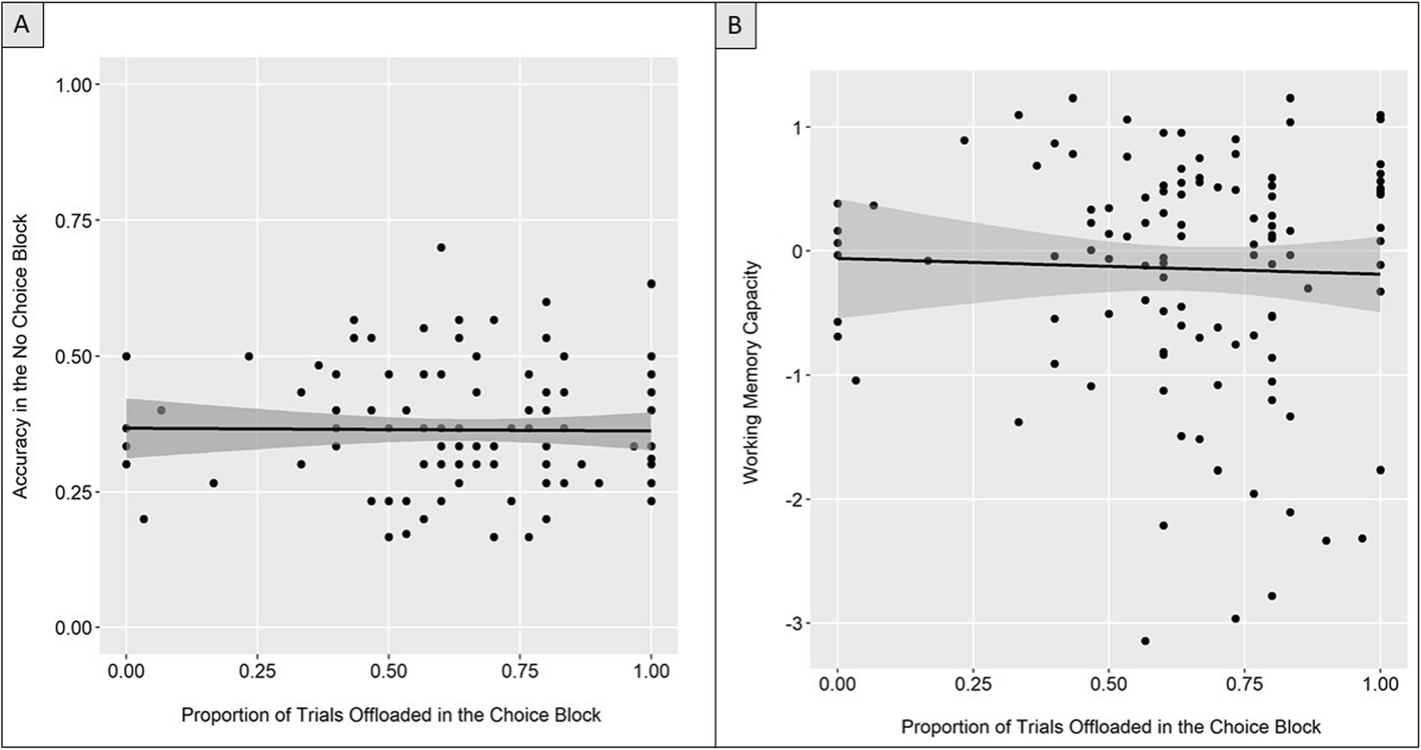
Scatter plots examining memory performance and offloading behavior. **a** Performance on the no-choice block and frequency of offloading. **b** Working memory, capacity, indexed by a composite of performance on complex working memory span tasks, and frequency of offloading. Errors bands represents the 95% confidence interval.

Next, following the analysis of Risko and Dunn (2015), we correlated performance in the no-choice block with the frequency of offloading in the choice block. We predicted that better performance in the no-choice block would be inversely related to offloading frequency (i.e., better internal memory related to less offloading, poorer internal memory related to more offloading), as was reported by Risko and Dunn (2015). We failed to find support for the hypothesized pattern (*r* (112) = −0.012, *p* = .897; Fig. 4).

### Short-term and working memory performance and offloading choice behavior

Next, we tested whether WMC explains variance above and beyond what is explained by no-choice block performance. Prior to conducting the regression we conducted an analysis designed to check for suppression in the planned regression and did not find evidence of suppression.^3^ The initial model containing only performance on the no-choice block to predict offloading behavior did not provide a good fit (*R*^2^ < 0.001, adjusted *R*^2^ = −0.009, *F* (1, 112) = 0.017, *p* = .897). We expected that adding WMC to the model would improve explained variance in offloading behavior. Contrary to our prediction, the model containing both performance on the no-choice block and WMC did not predict offloading behavior (*R*^2^ = 0.001, adjusted *R*^2^ = −0.017, *F* (2, 111) = 0.066, *p* = .936) and did not provide a significantly better fit over the base model with no-choice block performance alone (*F* change (1, 111) = 0.116, *p* = .735).

### Choice behavior and working memory predicting offloading benefit

Last, we wanted to test what variable(s) best predicts the advantage of offloading information at high memory loads. Following a suppression check,^4^ we conducted a simultaneous regression predicting the benefit of offloading at high loads (performance in the choice block for high-load trials minus performance in the no-choice block for high-load trials) with the proportion of high-load trials offloaded and WMC. We expected that each of these predictors would account for unique variance in performance benefit in the choice block for high-load trials; specifically, we hypothesized that WMC would predict smaller differences in performance and that offloading frequency would predict larger performance differences in high-load trials across blocks. The overall model was significant (*R*^2^ = 0.148, adjusted *R*^2^ = 0.132, *F* (2, 111) = 9.619, *p* < .001). The proportion of high-load trials offloaded was a significant predictor in the model (*t* (111) = 4.349, *p* < .001), but WMC was not (*t* (111) = 0.821, *p* = .414).

## Discussion

In the current work we set out to elucidate the relationship between memory ability and offloading behavior, as well as the benefits to performance conferred by offloading. In a large sample of college students, we found support for the hypothesis that offloading benefits short-term memory performance at memory loads thought to be outside the capacity limits of short-term memory. On the other hand, our results failed to support the hypotheses that short-term memory and WMC are related to offloading frequency. This conclusion is supported by the absence of a significant short-term memory–offloading behavior relationship, representing a failure to replicate Risko and Dunn (2015), and the absence of a significant WMC–offloading behavior relationship, tested for the first time in this study. We also did not observe that WMC predicted the benefits of offloading to short-term memory performance at high memory loads.

These null results are surprising, not only due to the fact that they represent a failure to replicate and extend an effect reported by Risko and Dunn (2015) and similar findings in the prospective memory literature (Gilbert, 2015a; cf. Boldt & Gilbert, in press), but also because it is sensible to expect that individuals with poorer memory abilities should offload more than those with better memories. Risko and Dunn (2015) were sufficiently cautious in their initial reporting of the correlation between short-term memory performance and offloading behavior given the small sample size used in their study. Still, we had expected to replicate this pattern due to the prior literature (e.g., Gilbert, 2015a; Risko & Dunn, 2015) coupled with expectations about sensible relationships between these variables. However, a recently published report examining prospective memory also failed to find a significant relationship between performance under conditions during which internal memory only could be used and offloading behavior (Boldt & Gilbert, in press). In light of this result, the authors suggested that corrective feedback may be a critical factor in supporting the relationship between accuracy in internal memory conditions and offloading behavior (Boldt & Gilbert, in press). Unfortunately, the pattern of results obtained here did not allow us to shed any light on the central question regarding for whom offloading might be most beneficial; instead, we find support for a pattern that suggests that offloading confers performance benefits regardless of one’s internal memory ability.

In considering the differences between our dataset and that reported by Risko and Dunn (2015), we note a few points of divergence. First, performance on the auditory short-term memory task in our sample is overall lower than that reported in the sample obtained by Risko and Dunn (2015). Importantly, performance in our sample tracks sensible patterns (e.g., lower accuracy at higher memory loads, better overall performance in choice versus no-choice conditions) so we do not believe that this point of divergence represents lack of effort or lack of understanding in our sample. Instead, it is possible that differences in the student bodies, and therefore the sample of students enrolled in the departmental research pools, is an important factor to consider. Anecdotally, while all of those included in our final dataset reported fluency in English, a fair number of the students in our departmental pools are non-native English speakers. For example, the 2018–2019 academic year Psychology student subject pool at Stony Brook University (one of the data collection sites) had just over a quarter of the pool reporting a non-English language as their native language, and approximately half of the pool reported being fluent in two or more languages. However, over 97% of the participant pool reported being fluent in English. Therefore, it is possible that the inclusion of a large proportion of English-fluent non-native speakers in our final sample coupled with the use of single letters as stimuli (Guion, Flege, Akahane-Yamada, & Pruitt, 2000) may have made it particularly difficult for participants to correctly identify the presented letters during encoding. This could account for lower overall accuracy in our sample compared to Risko and Dunn (2015). However, we did observe increased offloading at higher memory loads, as well as evidence of the bimodal distribution in either choosing to write or not at each memory load as reported in Risko and Dunn (2015), suggesting that sample differences are unlikely to be the sole driving force behind our unexpected pattern of results. In addition to sample differences, we also note that participants in our study were only offered course credit as compensation for participating; Risko and Dunn (2015) offered participants either course credit or paid them for participation. Therefore, it is possible that there were motivational differences in the sample reported here compared to Risko and Dunn (2015) that may have contributed to overall differences in performance levels attained by each sample. Differences between the sample characteristics and compensation procedures used here compared to Risko and Dunn (2015), and their downstream effects on performance, are worthy of further exploration.

Another potential reason for the low performance exhibited by the sample reported here is tied to the auditory-only presentation of stimuli, following the procedure used by Risko and Dunn (2015). It is possible that participants in the current study may have encountered difficulty with phonologically similar letter perception, and unfortunately chose the wrong letter to report (and potentially offload). It is not clear why participants in the current study might be expected to have experienced more difficulty in this regard compared to the sample reported by Risko and Dunn (2015); nonetheless, the provision of corrective feedback and/or the addition of visual presentation to auditory stimulus presentation might have served to drive up performance in the sample reported here. Moreover, corrective feedback may have served to support the predicted relationship between internal memory and offloading behavior (as suggested by Boldt & Gilbert, in press); future studies to investigate the role of corrective feedback in guiding offloading behavior may serve to further clarify this relationship.

Why is it that we observed a benefit to memory for the no-choice block at set size 2, in contrast to the null effect at set size 2 for performance in the choice block versus the no-choice block reported by Risko and Dunn (2015)? Although this pattern was not predicted a priori, we speculate that there may in fact be some small cost to external attention when one chooses to offload, which is far outweighed by the benefits to performance at larger set sizes. However, at very small set sizes, where offloading benefits are less likely to occur, there may only be costs to performance. Additional confirmatory research on this particular point may be of use to understand the durability of the pattern observed here.

Perhaps more directly relevant to offloading behavior, why would participants choose to write information down in the absence of overt benefits to performance? The exact opposite pattern was observed at set size 2, and no benefits to performance from offloading were observed at set size 4. One intriguing possibility is that participants find it difficult or aversive to switch strategies on a trial-by-trial basis. In task-switching paradigms, switch trials are more difficult (responses are produced more slowly and are more error prone) than nonswitch trials (Monsell, 2003). In task-switching paradigms that allow participants to choose whether to switch tasks or not rather than relying on external cues to indicate switch trials, participants sometimes report that they had intended to switch tasks, but that once the trial started they found themselves unprepared to perform the new task and therefore perform the previous task as that on the prior trial (see Arrington, 2017), suggesting some disconnect between intention and action. Future studies designed to directly address the potential disconnect between intentions and executed action in the context of offloading will provide important insights into the metacognitive processes related to offloading behavior.

Another question that remains is why errors might *ever* be observed on trials that are offloaded. Given the opportunity to write information down, one might expect ceiling level performance at all memory loads. However, this effect was not obtained here, nor by Risko and Dunn (2015). There are at least two potential error sources for offloaded trials: 1) incorrect/incomplete information offloaded; and 2) incorrect reporting of correctly offloaded information. Risko and Dunn (2015) report a larger proportion (approximately two-thirds) of errors attributed to incorrect or incomplete copying, and about one-third of errors were isolated to reporting errors. When incorrect/incomplete copying was found to be the source of the error, participants reported what was written about 50% of the time. Unfortunately, we are unable to isolate the source of errors here under the current method and can therefore provide no additional insight on this front. Future studies should be undertaken to examine the source of errors on offloaded trials, and perhaps the relationship of these sources to other cognitive and metacognitive abilities.

Returning to the question regarding for whom offloading is most beneficial, we were unable to provide answers to this question herein given the lack of support for relations between short-term memory and WMC and offloading (both offloading behavior and benefits conferred by offloading). These data prompt the question about other individual difference factors that might be better predictors of choice behavior and benefit from offloading. Importantly, these need not be predicted by the same factors. For example, it may be the case that global assessments of memory (in)ability drives choice to offload (Dunn & Risko, 2016), but that a factor like attention to detail or conscientiousness might be a better predictor of the magnitude of the offloading benefit. So, someone who thinks they have a poor memory *and* is high on conscientiousness/attention to detail might choose to offload a good deal and offloads effectively by taking careful, correct note of the to-be-remembered information chosen for offloading. On the other hand, global assessments of good memory ability might lead one to choose to offload less overall, and being low in the personality trait of conscientiousness or tending to pay little attention to detail might make one particularly ineffective at offloading. To the latter, it is difficult to imagine how this factor might play a big role with simple, single-letter stimuli but could be a much more important predictor with more complex, real-world memoranda.

Although the failure to find support for the relationship between objective memory capacity and offloading behavior and the benefits from offloading was contrary to our predictions, this pattern is encouraging in terms of the potential for offloading as an effective strategy to improve performance of memory-based tasks. Many healthy older adults report that their memory abilities are not as good as they used to be (Dunlosky & Metcalfe, 2009), and memory impairment is the hallmark symptom associated with Alzheimer’s disease (Jahn, 2013). To the extent that the patterns observed in our younger adult population reported here hold across the lifespan, performance of memory-based activities in both healthy older adults and older adult populations with clinical memory impairment may stand to benefit from offloading. Importantly, the size of the benefit that these populations might expect to obtain does not appear to be tied to overt memory performance. While there are certainly practical limitations to the utility of offloading as a strategy to improve performance of memory-based tasks, such as when one is unable to access the offloaded information (e.g., Soares & Storm, 2017), objective memory ability does not appear to fall into this category. This is notable given that other memory improvement strategies, such as working memory training, tend to be more effective in individuals with higher objective memory ability to start with (Wiemers, Redick, & Morrison, 2018). Therefore, the lack of relation between the performance benefit from offloading and memory ability presents a unique opportunity for the use of offloading as an intervention strategy that might be beneficial for performance regardless of internal memory ability.

It is possible that offloading is beneficial because, compared to other training interventions and cognitive strategies aimed at improving memory performance, it is so simple to implement. Both training interventions and memory strategies typically require some level of instruction and practice in order to benefit performance. However, offloading appears to benefit performance without much instruction or practice, at least in healthy younger adults. It will be important to test the benefits of offloading for more complex, real-world stimuli, and in a larger variety of sample populations, to examine whether the fairly immediate and easy-to-obtain benefits of offloading observed here are also evident in these other contexts.

## Conclusion

This report represents, to our knowledge, the first registered report focused on individual difference factors related to offloading behavior. As with many registered reports (Allen & Mehler, 2019) and preregistrations (e.g., Klein et al., 2018) we failed to find support for some of the previously reported patterns. We observed that short-term memory performance was unrelated to offloading choice as preliminarily reported by Risko and Dunn (2015), and that WMC was also unrelated to offloading choice behavior, contrary to our own hypotheses tested here for the first time. Moreover, we failed to find support for our own new prediction of an inverse relationship between WMC and benefit from offloading.

Overall, our findings serve to strengthen the idea that offloading does benefit performance and is particularly beneficial at higher memory loads. This pattern provides a direct replication of Risko and Dunn (2015). An important point of departure from findings reported in Risko and Dunn (2015) in the current study is the lack of support for a relationship between internal memory ability and both offloading choice and benefit from offloading. The pattern reported here is encouraging from the standpoint of prescribing the use of offloading as a strategy to rescue performance of memory-based activities in both healthy and cognitively compromised populations.

## Data Availability

Raw data and analysis script have been made available on the Open Science Framework Repository.

## Abbreviation

WMC: Working memory capacity

## Footnotes

1 A sample size of 114 also provides 81% power to detect an effect size of *f*^2^ = .09 for the regression described in question 4.

2 There is evidence that partial scoring methods are more sensitive to individual differences than absolute scoring methods (Conway et al., 2005), and thus may be a suitable approach to the current set of research questions. As such, partial credit scoring was also conducted where participants were awarded credit for each correct letter in the correct position (for example, a participant who is presented with the letters “QHLBXT” but reports “QDLBXT” would be awarded a score of 5 out of the possible 6 rather than scoring the entire trial as incorrect). Visual inspection of data and plots suggested that patterns were similar across scoring methods. Thus, only absolute scoring, the method used by previous authors and pre-specified herein, was used for statistical testing and is reported here.

3 Briefly, we conducted two bivariate regressions between combined performance on the working memory span tasks and the proportion of trials offloaded in the choice block, and performance on the no-choice block and the proportion of trials offloaded. We compared these data to partial correlations controlling between no-choice block performance and proportion of trials offloaded controlling for WMC, and between proportion of trials offloaded and WMC controlling for performance in the no-choice block. If the partial correlations were found to be larger by absolute value than the bivariate correlations this would have provided evidence for suppression among these variables. Fortunately, this was not observed (WMC and offloading bivariate *r* = −0.034, WMC and offloading controlling for no-choice performance *r* = −0.032; no-choice performance and offloading bivariate *r* = −0.012, no-choice performance and offloading controlling for WMC *r* = −0.004).

4 Suppression check: Before moving on to testing our last hypothesis, we performed another check for suppression amongst the variables we planned to include in the analysis. Again, we followed the rule that suppression would be evident should the absolute value of the partial correlation be larger than that of the bivariate correlation that includes the same variables without the control variable included. We correlated the offloading proportion on high load trials only (6, 8, and 10 items) with WMC, and a second bivariate correlation relating proportion of high-load trials offloaded with accuracy on high-load trials in the no-choice block was tested. Here, the complimentary partial correlations are the former controlling for accuracy on high-load trials in the no-choice block, and the latter controlling for WMC. We see no evidence for suppression among these variables (WMC and high-load offloaded: *r* = −0.057, WMC and high-load offloaded controlling for no-choice block performance on high-load trials: *r* = −0.026; high-load trial accuracy in the no-choice block with proportion of high-load trials offloaded in the choice block: *r* = −0.090, high-load trial accuracy in the no-choice block with proportion of high-load trials offloaded in the choice block controlling for WMC: *r* = −0.074).
